# Supplementary material for: Association between oral microbiome and seven types of cancers in East Asian population: a two-sample Mendelian randomization analysis
Source: Front Mol Biosci. 2023 Nov 21;10:1327893. doi: 10.3389/fmolb.2023.1327893 (PMC10702768; doi:10.3389/fmolb.2023.1327893)
Supplement: Supplementary file 1 [file DataSheet1.ZIP › Supplementary Materials/MR plots of saliva/saliva═╝/Prostate cancer/pheno.2370_to_prostate cancer_forest.pdf]

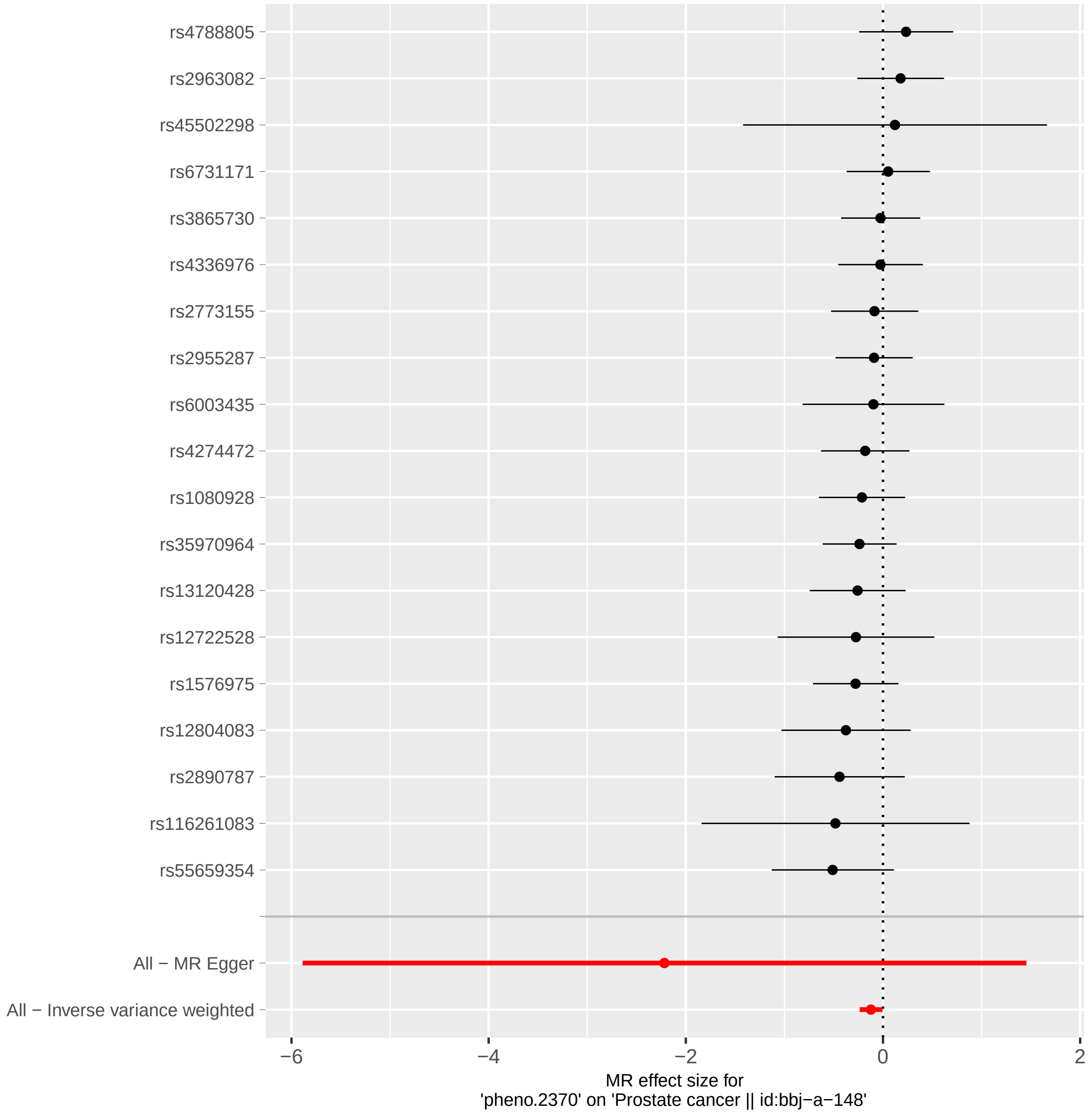
rs4788805
rs2963082
rs45502298
rs6731171
rs3865730
rs4336976
rs2773155
rs2955287
rs6003435
rs4274472
rs1080928
rs35970964
rs13120428
rs12722528
rs1576975
rs12804083
rs2890787
rs116261083
rs55659354
All − MR Egger
All − Inverse variance weighted
−6
−4
−2
0
2
MR effect size for
'pheno.2370' on 'Prostate cancer || id:bbj−a−148'
